# Comparing external fixators and intramedullary nailing for treating open tibia fractures: a meta-analysis of randomized controlled trials

**DOI:** 10.1186/s13018-022-03490-x

**Published:** 2023-01-05

**Authors:** Jun Liu, Lifeng Xie, Li Liu, Guicheng Gao, Ping Zhou, Dejun Chu, Dewei Qiu, Jun Tao

**Affiliations:** 1 Department of Orthopedics, The Second Affiliated Hospital of Nanchang University, No. 1 Minde Road, Nanchang, 330006 Jiangxi Province China; 2 Department of Gastrointestinal Surgery, The First Affiliated Hospital of Nanchang University, Nanchang, China

**Keywords:** Open tibia fractures, Tibia fractures, External fixators, Intramedullary nailing, Meta-analysis

## Abstract

**Background:**

External fixators (EFs) and intramedullary nailing (IMN) are two effective methods for open tibial fractures. However, both methods have advantages and disadvantages, and the optimal surgical approach remains controversial. Thus, we performed a meta-analysis of randomized controlled trials (RCTs) to compare EF with IMN to evaluate their efficacy and safety.

**Methods:**

A systematic study of the literature was conducted in relevant studies published in PubMed, Embase, the Cochrane Library, Web of Science, CNKI, CBM, Wanfang and Weipu from database inception to April 2022. All eligible literature was critically appraised for methodological quality via the Cochrane's collaboration tool. The primary outcome measurements included postoperative superficial infection, postoperative deep infection, union time, delayed union, malunion, nonunion, and hardware failure.

**Results:**

Nine RCTs involving 733 cases were included in the current meta-analysis. The pooled results suggested that cases in the IMN group had a significantly lower postoperative superficial infection rate [risk ratio (RR) = 2.84; 95% confidence interval (CI) = 1.83 to 4.39; *P* < 0.00001)] and malunion rate (RR = 3.05; 95% CI = 2.06 to 4.52; *P* < 0.00001) versus EF, but IMN had a significantly higher hardware failure occurrence versus EF (RR = 0.38; 95% CI = 0.17 to 0.83; *P* = 0.02). There were no significant differences in the postoperative deep infection rate, union time, delayed union rate or nonunion rate between the two groups (*p* > 0.05).

**Conclusions:**

Compared to EF, IMN had a significantly lower risk of postoperative superficial infection and malunion in patients with open tibial fractures. Meanwhile, IMN did not prolong the union time and increased the risk of the deep infection rate, delayed union rate and nonunion rate but had a higher hardware failure rate. The reanalysis of union time showed that it was significantly shorter in the IMN group than in the EF group after excluding the study with significant heterogeneity during sensitivity analysis. Therefore, IMN is recommended as a preferred method of fracture fixation for patients with open tibial fractures, but more attention should be given to the problem of hardware failure.

**Supplementary Information:**

The online version contains supplementary material available at 10.1186/s13018-022-03490-x.

## Introduction

Open tibial fracture is the most common type of open fracture of the long bones of extremities and is most commonly seen in traffic accidents [1–3]. For patients with open tibial fracture, emergency debridement of wounds, vascular and nerve exploration, early soft tissue coverage and stabilization of fractures are agreed upon treatments [4]. Among them, the two most commonly used surgical methods to fix the fracture are external fixators (EF) and intramedullary nailing (IMN). However, both methods have their own advantages and disadvantages, and which one is better is still controversial. In the 1990s, EF was widely used in open fractures due to its advantages of rapid operation, no surgical incision and no influence on blood supply to the fracture site [5]. However, postoperative patients with EF often suffer from complications, such as needle path infection, fracture malunion, reduction loss and joint contracture [6, 7]. In addition, the long-term use of EF also causes great inconvenience to the nurses of needles and the life of patients. Currently, in the treatment of open tibial fractures, IMN has been widely used because of its advantages of central fixation, early weight-bearing, minimal invasiveness and convenient postoperative care [8, 9], but there are also risks of hardware failure and infection diffusion through the medullary cavity [10].

In view of the above controversies, some scholars have performed meta-analyses on the treatment of open tibial fractures with IMN and EF, but there have been limitations. Fu et al. [11], for example, did not compare the fracture healing time between the two groups in the outcomes of the meta-analysis. The meta-analysis conducted by Xu et al. [12] did not conduct heterogeneity analysis on outcomes with significant heterogeneity, such as union time, to explore the source of heterogeneity. In the meta-analyses of Zhang et al. [13] and Fang et al. [14], some of the included studies were retrospective studies and case reports, which undoubtedly affected the level of evidence. In addition, Garg et al. [15], Kyengera et al. [16] and Haonga et al. [17] recently reported a randomized clinical trial (RCT) study on the treatment of open tibial fractures with EF and IMN. The inclusion of these studies may have changed the results of similar studies described above.

In view of the above problems, the purpose of this study was to collect all available RCTs on the treatment of open tibial fractures with EF and IMN for meta-analysis to provide reliable evidence-based medical evidence for clinical decision-making.

## Materials and methods

### Search strategy

A systematic electronic search of databases such as PubMed, EMBASE, Cochrane Library, Web of Science, CBM, CNKI, Wanfang and Weipu was conducted to identify published studies from inception till April 2022. Also, the manual search was performed through checking the reference lists of key studies and review articles to identify additional studies. Search terms are including "tibial fractures," "intramedullary nail," "external fixators," "fracture fixation," and "randomized controlled trial" were used individually or combined using the Boolean operators “AND” or “OR”. The publishing language was confined to Chinese and English.

### Selection criteria

Studies were considered eligible by two reviewers with a background in orthopedics independently when they met following criteria: (1) patients who were skeletally mature with open tibial fracture, and studies must have had two or more groups where one of them must have used EF and another IMN to fix the tibial fracture; (2) published clinical RCTs; (3) studies with at least one of the following outcomes: postoperative superficial infection, postoperative deep infection, union time, delayed union, malunion, nonunion and hardware failure. Studies would be excluded for duplicates, conference abstract or thesis, animal or biomechanical studies, case report or review, follow-up less than 12 months and full text unavailable. Any controversy was resolved with discussion between the reviewers or to consult a third reviewer.

### Quality assessment and data extraction

To evaluate inclusion eligibility, a quality assessment tool of "Cochrane Collaboration’s tool for assessing risk of bias" was used, which recommended in the Cochrane Handbook (version 5.1.0, updated March 2011) that includes six major possible sources of bias: random sequence generation, allocation concealment, blinding of participants and personnel, incomplete outcome data, selective reporting and anything else [18]. The same two reviewers extracted the following data using a spreadsheet from each eligible study: first author’s name, publication year, country, patients’ age and gender, number of participants, follow-up duration, materials of fixator, fracture types (Gustilo-Anderson classification [19]) and the above-mentioned outcomes of interest. When relevant data were missing or unclear, the study authors will be contacted. Any discrepancies in results were resolved with discussion between the reviewers or to consult a third reviewer.

### Data analysis

Data from included studies were analyzed with Review Manager software (Version 5.3. Copenhagen: The Nordic Cochrane Centre, The Cochrane Collaboration, 2014). Dichotomous variables, such as postoperative superficial infection, postoperative deep infection, delayed union, malunion, nonunion and hardware failure, were expressed by risk ratio (RR) and 95% confidence interval (CI), while continuous outcomes, such as union time, were summarized by the mean difference (MD) and 95% CI. If continuous data were reported with mean and ranges, the standard deviations were calculated to use a special method recommended in the Cochrane Handbook [20]. Statistical heterogeneity will be determined by I-square test [21], when I2 is higher than 25%, 50%, and 75%, the heterogeneity is low, moderate, and high, respectively [22]. When *I*^2^ < 50%, a fixed-effects model will be used. On the contrary, a random-effects model will be selected. To assess sources of heterogeneity, sensitivity analysis or subgroup analyses was conducted. A *P* value of < 0.05 was considered to be statistically significant for all analyses.

## Results

### Search results

**Fig. 1:**
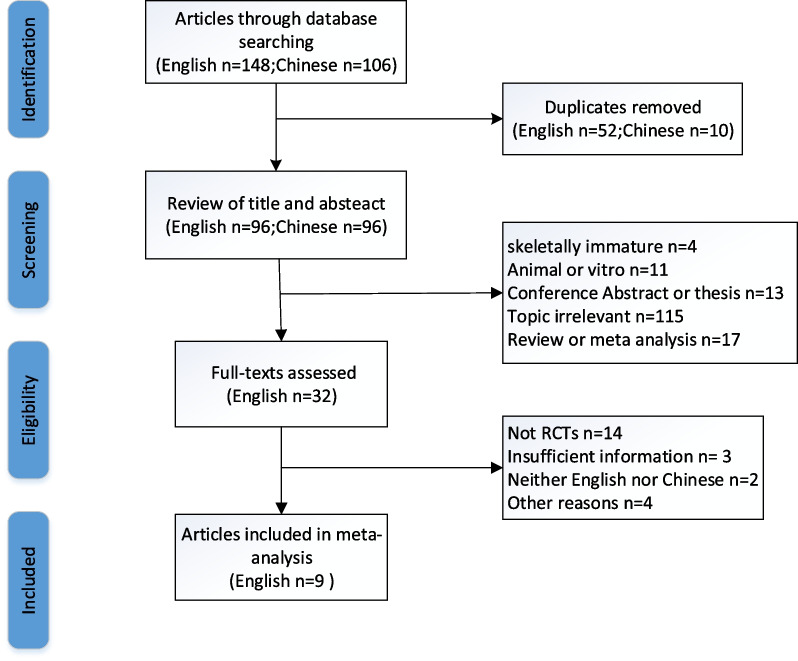
Flowchart of study selection process

A total of 254 potentially relevant citations were extracted from the eight electronic databases. After removing duplicates and reading the abstract and title, 32 studies were screened for relevance. Eventually, nine RCTs [6, 15–17, 23–27] with 733 cases (346 EF and 387 IMN) were considered to meet the eligibility criteria and included in the meta-analysis after screening the full-text, The included studies were published during the period 1989 and 2022. A flowchart of the study selection process is illustrated in Fig. 1

### Characteristics of eligibility studies

Most of the included studies reported the content of the author information, publication year, study population characteristics (e.g., age, sex, and nation), number of participants, follow-up duration, materials of fixator, fracture types. There are four eligible studies were conducted from Asian countries, three from the USA and two from African countries. The average age varied from 25 to 41 years old and sample size ranged from 14 to 104, and with 539 male patients and 131 female patients except the study of Holbrook et al. Duration of follow-up ranged from 4.5 to 46.5 months. In addition, the materials of fixator, fractures classified by Gustilo classification and a summary of the basic characteristics are listed in Table 1.

**Table 1:** The characteristics of included studies

Author	Country	Age (mean, years)	Gender (male/female)		Sample size	Follow-up (mean, month)	Materials		GA classification
		IMN/EF	IMN	EF	IMN/EF	IMN/EF	IMN	EF	
Holbrook [23]	USA	28/25	NA	NA	29/28	16.8/18.5	Ender	half-pin	I, II, III
Tornetta [24]	USA	41/37	11/4	9/5	15/14	21^b^	Gross-Kempf, Alta and AO	Hoffman and Ace	IIIB
Tu [25]	Taiwan	38.5^a^	30/6^c^		18/18	20.5^b^	Russell-Taylor and AO	Hoffmann	IIIA, IIIB
Henley [26]	USA	33/33	79/21	53/15	104/70	15.7/17.6	NA	half-pin	II, IIIA, IIIB
Inan [6]	Turkey	31.7/32.3	24/5	28/4	29/32	43.3/46.5	Russell-Taylor, Synthesnails and Orthofixnails	Ilizarov	IIIA
Mohseni [27]	Iran	30.8/28.92	20/5	22/3	25/25	12^b^	NA	AO tubular plate	IIIA, IIIB
Garg [15]	India	40.44/38.76	18/7	19 /6	25/25	36^b^	NA	Half-Pin	IIIA, IIIB
Haonga [17]	Tanzania	33.3/31.8	98/13	91/19	111/110	12^b^	SIGN	AO uniplanar Dispofix	I, II, III
Kyengera [16]	Uganda	39/39	21/10	16/8	31/24	12/4.5	NA	NA	II, IIIA

*USA* the United States of America, *IMN* intramedullary nail; EF: external fixation, *NA* not available, *GA* Gustilo–Anderson

^a,b^Mean age and follow-up of patients was included, respectively, regardless of IMN or EF

^c^Gender of patients was included, regardless of man or female

### Quality assessment of the eligible studies

**Fig. 2:**
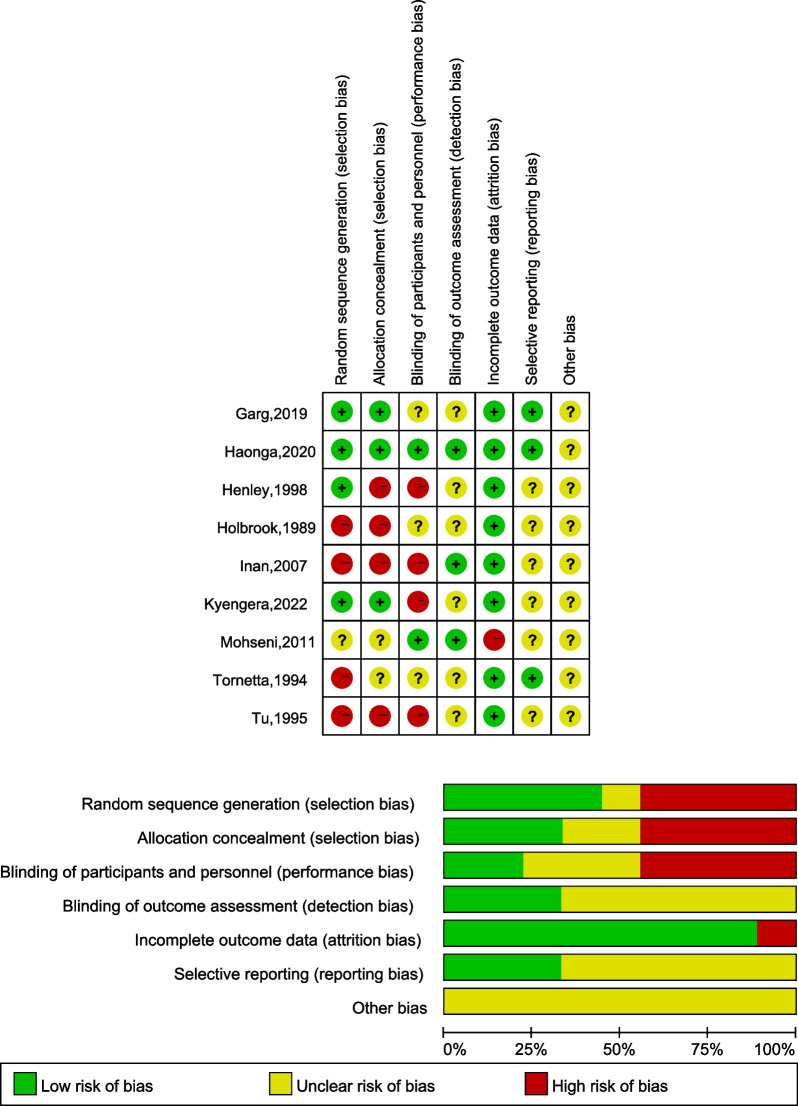
Risk of bias graph and Risk of bias summary

Nine RCTs [6, 15–17, 23–27] were assessed by the Cochrane Handbook, the detailed information of which is illustrated in Fig. 2. All reported the method of randomization. Two studies conducted by Kyengera et al. [16] and Garg et al. [15] were randomized by opaque envelopes were prepared onsite and randomized chit box, respectively. Five studies [6, 23–26] mentioned that the randomization was realized based on even/odd the hospital medical-record number of the patient. Two studies [15, 27] did not describe the method of concealing group allocation. Another, the study conducted by Mohseni et al. [27] pointed out that follow-up was done by an observer blind to the group of the patients in their study. All studies showed a Low risk of bias due to incomplete outcome. Five studies [6, 16, 25–27] showed an unclear bias due to selective outcome reporting.

### Results of meta-analysis

#### Postoperative superficial infection

**Fig. 3:**
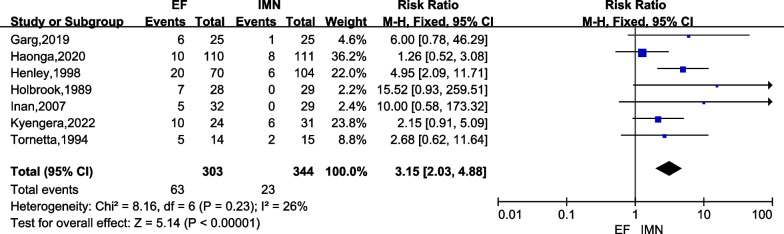
Forest plots for comparing the postoperative superficial infection between EF and IMN

Seven studies [6, 15–17, 23, 24, 26] with a total of 647 cases (EF = 303, IMN = 344) provided data on postoperative superficial infection. There was low heterogeneity among these studies (*I*^2^ = 26%), and the fixed-effects model was used. The meta-analysis showed that the IMN group had significantly lower superficial infection versus the EF group (*RR* = 3.15; 95% CI = 2.03 to 4.88; *P* < 0.00001) (Fig. 3).

#### Postoperative deep infection

**Fig. 4:**
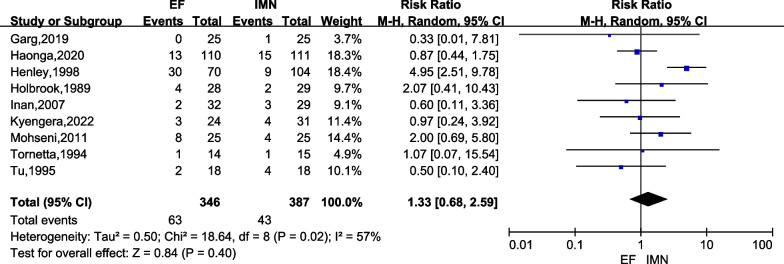
Forest plots for comparing the postoperative deep infection between EF and IMN

Nine studies [6, 15–17, 23–27] comprising of 733 cases (EF = 346, IMN = 387) in both groups reported on postoperative deep infection. There was moderate heterogeneity among the studies (*I*^2^ = 57%). Data were pooled using a random-effects analysis, and the meta-analysis indicated that there was no significant difference in deep infection occurrence between IMN and EF groups (*RR* = 1.33; 95% CI = 0.68 to 2.59; *P* = 0.40) (Fig. 4).

#### Union time

**Fig. 5:**
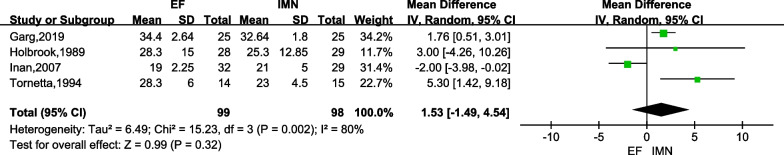
Forest plots for comparing the union time between EF and IMN

Four studies [6, 15, 23, 24] containing 197 cases (EF = 99, IMN = 98) in both groups stated the union time, and the high heterogeneity among studies indicated a random-effect model should be adopted (*I*^2^ = 80%). The meta-analysis showed that there was no significant difference in union time between IMN and EF groups (*MD* = 1.53; 95%*CI* = -1.49 to 4.54; *P* = 0.32) (Fig. 5).

#### Delayed union

**Fig. 6:**
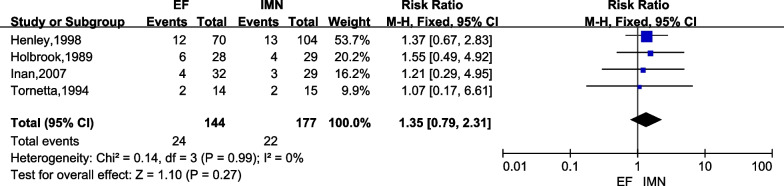
Forest plots for comparing the delayed union between EF and IMN

Four studies [6, 23, 24, 26] of 321 cases (EF = 144, IMN = 177) reported the incidence of delayed union. There was no significant difference between IMN group and EF group according to the meta-analysis with fixed-effect model (*I*^2^ = 0%). Figure 6 lists the result of delayed union in both groups (*RR* = 1.35; 95%*CI* = 0.79 to 2.31; *P* = 0.27).

#### Malunion

**Fig. 7:**
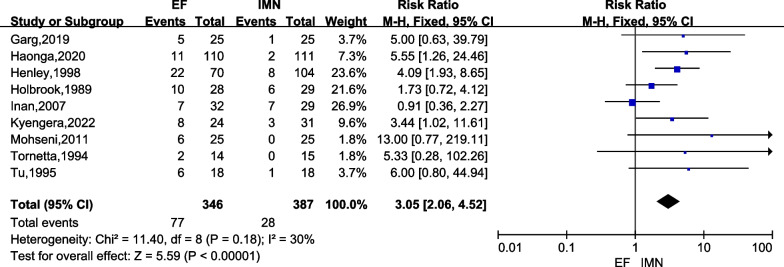
Forest plots for comparing the malunion between EF and IMN

Malunion was reported in all studies [6, 15–17, 23–27], and the data were extracted since low heterogeneity was found (*I*^2^ = 30%). The malunion rate was analyzed by a fixed-effects model and we found the IMN group had significantly lower malunion rate versus the EF group (*RR* = 3.05; 95% *CI* = 2.06 to 4.52; *P* < 0.00001) (Fig. 7).

#### Nonunion

**Fig. 8:**
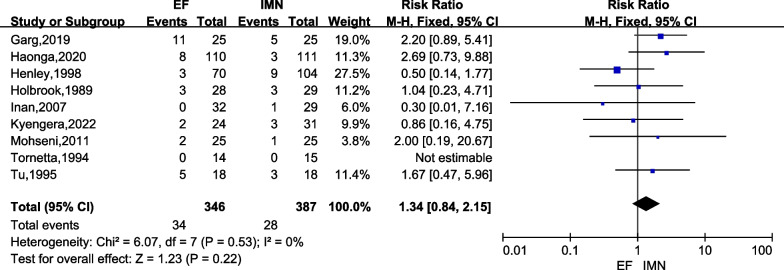
Forest plots for comparing the nonunion between EF and IMN

Nonunion were presented in all studies [6, 15–17, 23–27]. There was no heterogeneity among these studies (*I*^2^ = 0%), and the fixed-effects model was used. The present meta-analysis revealed that there was no significant difference in nonunion rate between IMN and EF groups (*RR* = 1.34; 95% CI = 0.84 to 2.15; *P* = 0.22) (Fig. 8).

#### Hardware failure

**Fig. 9:**
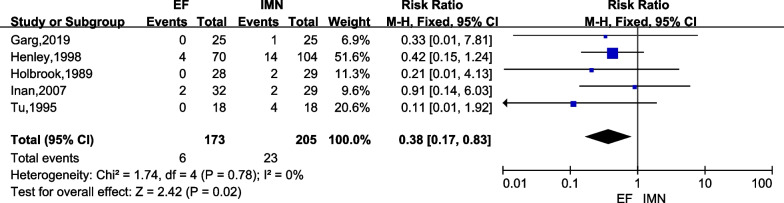
Forest plots for comparing the fixation failure between EF and IMN

Hardware failure was reported in five studies [6, 15, 23, 25, 26] focusing on 378 cases (EF = 173, IMN = 205). There was no heterogeneity (*I*^2^ = 0%), therefore, a fixed-effects model was adopted. The result of meta-analysis indicated that the IMN group had significantly higher the incidence of hardware failure versus the EF group (*RR* = 0.38; 95% CI = 0.17 to 0.83; *P* = 0.02) (Fig. 9).

## Publication bias

**Fig. 10:**
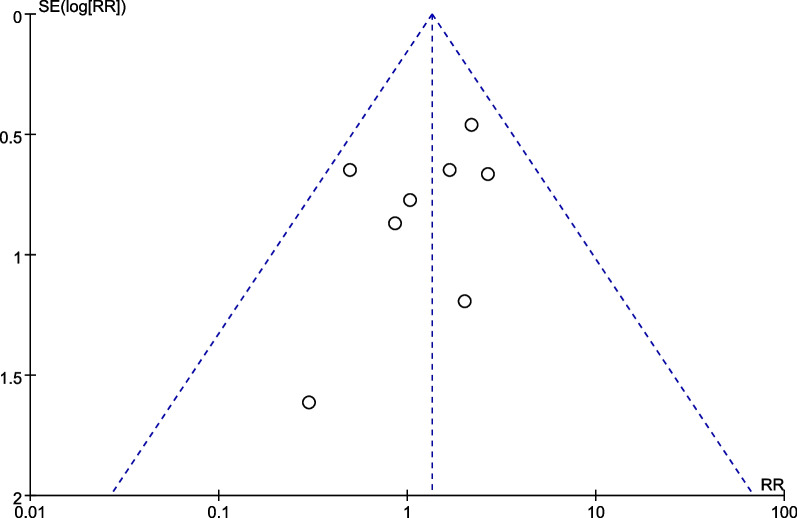
A funnel plot of nonunion

Publication bias was assessed by a funnel plot diagram. The funnel plot diagrams of nonunion were symmetrical, indicating a low risk of publication bias (Fig. 10).

## Sensitivity analysis

To investigate the sources of heterogeneity, sensitivity analysis was performed by excluding each study sequentially based on the results of postoperative deep infection and union time. For pooled analysis on postoperative deep infection, the results showed that after excluding the studies conducted by Henley et al. [26], heterogeneity was disappeared (*I*^2^ = 0%) (Additional file 1). For assessment on union time, the heterogeneity reduced from *I*^2^ = 80% to 32% after excluding the studies conducted by Inan et al. [6] (Additional file 2). In addition, we also used different effect models to assess the stability of the results. The sensitivity analysis of postoperative superficial infection, delayed union, malunion, nonunion, and hardware failure were consistent whether using a fixed‐effects model or a random‐effects model.

## Discussion

The purpose of this study was to evaluate and compare the efficacy and safety of EF and IMN in the treatment of open tibial fractures. The results showed that the incidence of postoperative superficial infection and malunion in the IMN group was significantly lower than that in the EF group, while the IMN group had a significantly higher IMN hardware failure rate than the EF group. There were no significant differences in the postoperative deep infection rate, union time, delayed union rate or nonunion rate between the two groups.

The study of Gristina et al. [28] showed that the presence of human implants would lead to a corresponding increase in the infection rate, which may be caused by the difficulty of the immune system to eliminate bacteria residing on the surface of inactive implants. In our study, the superficial infection rate in the EF group was significantly higher than that in the IMN group, which was consistent with the study of Fu et al. [11] and Zhang et al. [13]. Most of the superficial infections in the EF group occurred in the pin tracks, while most of the infections in the IMN group occurred at the surgical incision, which may be related to the difficulty of effective nursing of the EF pin-tract. Of note, the study of Haonga et al. [17] showed that superficial infections in the EF group did not include pin-tract-related infections, which may have biased the results to some extent.

According to the study of Rohde et al. [10], the treatment of open tibial fractures with IMN has the risk of infection spreading along the medullary cavity, and in his study, IMN had a higher incidence of osteomyelitis than EF. However, this study showed that there was no significant difference in the deep infection rate between EF and IMN, which was consistent with previous studies [11, 13, 14]. Another recent study [3, 4] reported that antibiotic-coated nails can significantly reduce the risk of infection. Henley et al. [26] reported that, for patients with open tibial fractures, the severity of soft tissue and bone damage or contamination has a greater impact on the occurrence of postoperative infection than the choice of fixation method. The study of Li et al. [29] also showed that the incidence of infection in patients with open tibial fracture was related to the severity of fracture, whether debridement was complete, early soft tissue coverage, smoking and other factors. Therefore, the choice of fracture fixation method is not a principal factor leading to deep infection after open tibial fracture.

However, there was moderate heterogeneity in the deep infection rate among the included studies, which was found to be derived from the study of Henley et al. [26] after sensitivity analysis. When this study was excluded, the heterogeneity disappeared (*I*^2^ = 57% to 0%). The reasons for the heterogeneity may be as follows: on the one hand, in the study of Henley et al., antibiotics were used for a short period of time and were only administered perioperatively to all patients for twenty-four to forty-eight hours, which may be an important factor leading to the high infection rate; on the other hand, he classified all postoperative wound problems requiring intravenous antibiotics as deep infections regardless of the duration of intravenous antibiotics, which would undoubtedly lead to an increase in the number of deep infections.

Our study showed no significant difference in union time between EF and IMN. However, there was high heterogeneity in the union time among the studies, and sensitivity analysis revealed that heterogeneity came from the study of Inan et al. [6]. When this study was excluded, it was found that heterogeneity decreased to a low level (*I*^2^ = 80% to 32%); moreover, the results of the reanalysis showed that union time was significantly shorter in the IMN group than in the EF group (*MD* = 2.12; *95% *CI = 0.94 to 3.29; *P* = 0.0004) (Additional file 2). This is consistent with the results of previous similar studies [12, 13]. In general, IMN is more stable for fracture fixation and has fewer superficial infection events than EF, both of which are beneficial for fracture healing. In addition, union time is also related to surgeons' techniques, the health condition of patients, skin and soft tissue contamination and other factors [30].

In the study of Inan et al. [6], we believe that the following reasons may lead to high heterogeneity: first, in his study, the age of patients included in the EF group and IMN group was younger, with an average age of 32.3 and 31.7 years, respectively, which may have led to relatively fast fracture healing; second, Inan used a different definition of fracture healing in the study; finally, it may be related to the patient's postoperative rehabilitation management and surgical techniques.

Malunion was defined as varus or valgus malalignment of 5 degrees or more, anterior or posterior angulation of 10 degrees or more, shortening of one centimeter or more, or rotational malalignment of 10 degrees or more compared with the contralateral leg [23]. This meta-analysis showed that the malunion rate in the IMN group was significantly lower than that in the EF group, which was consistent with the findings of Donnelley et al. [31]. On the one hand, although the EF method has the advantages of simple operation and low cost, it also has the disadvantage of insufficient exposure of the fracture site, which will lead to difficulty in accurate reduction [14]. At the same time, because of its inherent characteristics, EF has difficulty maintaining a good reduction state of the fracture until bone healing is achieved. On the other hand, IMN can be inserted into the tibial medullary cavity to function as internal splints, which can firmly fix the fracture [32] and reduce the incidence of malunion. In addition, once the healing procedure begins, regardless of the alignment condition, final bone healing will be achieved [14]. This study showed that there was no significant difference in the delayed union rate and nonunion rate between the EF group and the IMN group, which was consistent with the study of Schandelmaier et al. [33].

Hardware failures were defined as EF pin breakage and IMN nail or locking screw breakage. This meta-analysis showed that the IMN group had a significantly higher hardware failure rate than the EF group. A study by Whittle et al. [34] on the application of small-diameter IMN in the treatment of tibial fractures showed that hardware failure occurred in 13.8% of tibial fractures, most of which came from locking screws, and pointed out that hardware failure was highly correlated with delayed union. Similar to other metallic fracture fixation implants, similar studies [32, 35] also pointed out that IMN may eventually lead to fatigue breakage if bone healing does not occur, and the breakage of the distal locking nail is the most common complication. The study of Lin et al. [36] further pointed out that the closer the fracture site is to the distal locking screw, the less contact of nail-cortical, and the smaller number of locking screws will lead to increased stress on the locking screw and increase the risk of hardware failure.

This meta-analysis is an update of previous similar research and has some limitations. First, due to the inconsistent evaluation criteria of patients' postoperative function in the included studies, this study was unable to analyze and compare the postoperative function of the two groups of patients. Second, not all of the included studies met strict randomization criteria, and some studies were assigned allocation concealment according to odd or even numbers of patients' case numbers. Finally, a small number of studies was included (< 10), resulting in low efficiency of funnel plot analysis and a high possibility of publication bias.

## Conclusions

In summary, compared with EF, IMN provides a lower postoperative superficial infection rate and malunion rate in patients with open tibial fractures. Meanwhile, IMN did not prolong the union time and increased the deep infection rate, delayed union rate or nonunion rate, in addition to having a higher hardware failure rate. It is worth pointing out that sensitivity analysis showed that union time was significantly shorter in the IMN group than in the EF group after excluding studies with significant heterogeneous sources.

Therefore, IMN is recommended as a preferred method of fracture fixation for patients with open tibial fractures. However, the extent of nail-cortical contact or the number of distal locking screws should be appropriately increased, or an IMN system with a higher elastic modulus should be selected to avoid hardware failure. In the future, high-quality RCTs are still required for further investigation.

## Supplementary Information

**Additional file 1**. Sensitivity analysis of deep infection.

**Additional file 2**. Sensitivity analysis of union time.

## Data Availability

Please contact the corresponding author for data requests.

## Abbreviations

EF: External fixators
IMN: Intramedullary nailing
RCT: Randomized clinical trial
RR: Risk ratio
CI: Confidence interval
MD: Mean difference
