# Supplementary figures and images for: Comparing external fixators and intramedullary nailing for treating open tibia fractures: a meta-analysis of randomized controlled trials
Source: J Orthop Surg Res. 2023 Jan 5;18:13. doi: 10.1186/s13018-022-03490-x (PMC9817243; doi:10.1186/s13018-022-03490-x)

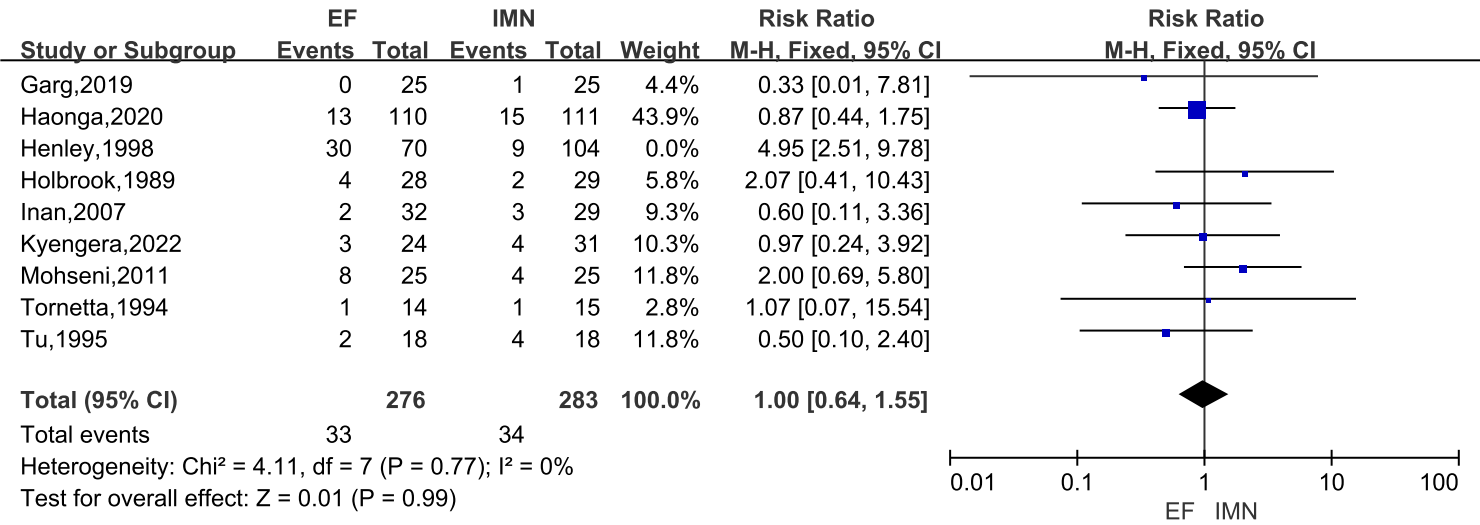

Supplement: Supplementary file 1 — Additional file 1. Sensitivity analysis of deep infection. [file 13018_2022_3490_MOESM1_ESM.pdf]

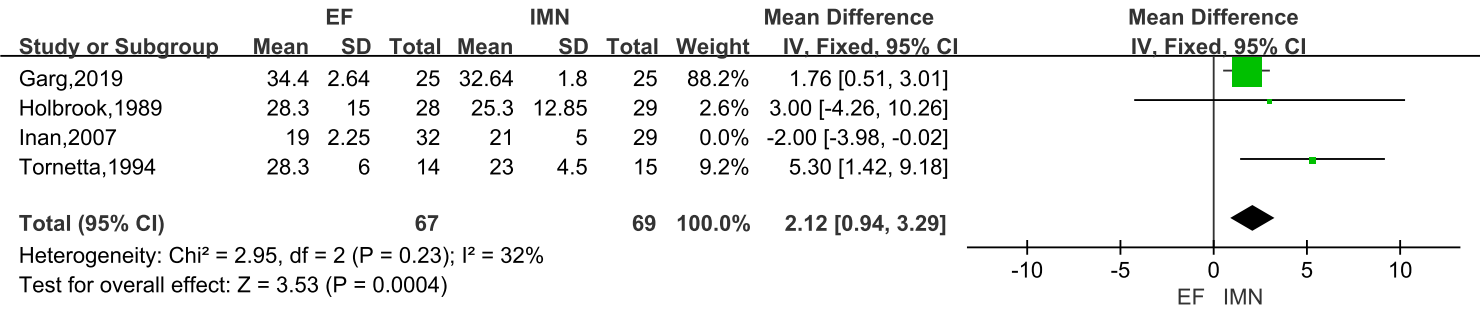

Supplement: Supplementary file 2 — Additional file 2. Sensitivity analysis of union time. [file 13018_2022_3490_MOESM2_ESM.pdf]
